# Ethnopharmacological survey of different uses of seven medicinal plants from Mali, (West Africa) in the regions Doila, Kolokani and Siby

**DOI:** 10.1186/1746-4269-1-7

**Published:** 2005-09-27

**Authors:** Adiaratou Togola, Drissa Diallo, Seydou Dembélé, Hilde Barsett, Berit Smestad Paulsen

**Affiliations:** 1 Section of Pharmacognosy, Department of Pharmaceutical Chemistry, University of Oslo PO Box 1068 Blindern, 0316, Norway; 2 Department of Traditional Medicine, BP 1746, Bamako, Mali

**Keywords:** Ethnopharmacology, Mali, *Opilia celtidifolia*, *Anthocleista djalonensis*, *Erythrina senegalensis*, *Heliotropium indicum*, *Trichilia emetica*, *Piliostigma thonningii*, *Cochlospermum tinctorium*

## Abstract

An ethnopharmacological survey was carried out to collect information on the use of seven medicinal plants in rural areas in the nearby regions of Bamako, Mali. The plants were *Opilia celtidifolia*, *Anthocleista djalonensis*, *Erythrina senegalensis*, *Heliotropium indicum*, *Trichilia emetica*, *Piliostigma thonningii *and *Cochlospermum tinctorium*

About 50 medical indications were reported for the use of these plants in traditional medicine. The most frequent ailments reported were malaria, abdominal pain and dermatitis. The highest number of usages was reported for the treatment of malaria (22%). The majority of the remedies were prepared from freshly collected plant material from the wild and from a single species only. They were mainly taken orally, but some applications were prepared with a mixture of plants or ingredients such as honey, sugar, salt, ginger and pepper. Decoction of the leaves was the main form of preparation (65%) and leaf powder was mostly used for the preparation of infusions (13%). The part of the plants most frequently used was the leaves. There was a high degree of informant consensus for the species and their medicinal indications between the healers interviewed.

The results of this study showed that people are still dependent on medicinal plants in these rural areas of Mali.

## Background

Located in West Africa, Mali is a landlocked country with an area of approximately 1, 246,000 Km^2 ^for an estimated population of 13 million inhabitants. Mali is one of the poorest countries in the world with a GPD of 725$ (2002) per capita [1]. The economy is essentially based on agriculture; the health sector policy promotes community-based, self-supported health care services and the administration of essential medicines including traditional medicines [2]. Like in many other developing countries, people in Mali use medicinal plants to improve their state of health. Traditional medicine is a significant element in the cultural patrimony. Its use has increased with the increase in price of conventional medicine in the local currency. Traditional medicine still remains the main recourse for a large majority of people for treating health problems. Approximately 80% of the population in Mali use traditional medicine as their only type of medicine [2]. Official medical attention is usually based on commercial drugs that have to be purchased with money, while a traditional medical consultancy has a much lower cost, including the consumption of the medicinal plants required [3]. Most of the plants used in this traditional medicine have never been investigated for their chemical composition and pharmacological activities. It is therefore important to study these plants to substantiate the traditional medical knowledge. People, and especially the traditional healers, should be informed of the benefits, risk and limitation of the plants they use for medical purposes.

A traditional healer is defined as a person with competence to practice traditional medicine. From 1968 to 1978 registration of traditional healers and medicinal plants was carried out in all the administrative regions in Mali by an interdisciplinary team. The competence of a healer is evaluated on the person's achievements on curing diseases and the results are essential for consideration of registering the person as a traditional healer. After being registered, the DMT sets up a principle of collaborating with the traditional healers. The collaborating healer is not obliged to deliver samples of his medications to DMT, but if he wishes to do so the plants will be subjected to toxicological, pharmacological and phytochemical analyses, the results of which are given to the healer. As a result of this collaboration, the healer is granted official recognition as a practitioner in traditional medicine and is provided with an identity card for traditional practitioners. Other traditional healers are also allowed to practice with no restriction, but they do not have a registration card. In some localities of Mali, the healers are grouped in association and have created gardens of medicinal plants [2]. All studies being undertaken between DMT and the traditional healers follow ethical aspects and rules set down by the local government as both DMT and the traditional healers are part of the health care system of Mali.

The Department of Traditional Medicinal (DMT), the first research establishment for the study of medicinal plants in Mali, and a collaborating centre of World Health Organisation (WHO) on traditional medicine, has as the main objectives: the registration of traditional healers, traditional knowledge and medicinal plants, in addition to perform research, and to develop Improved Traditional Medicines (ITMs) from local plants.

Several medicinal plants have been studied in the laboratory of DMT using classical methodology for phytochemistry, pharmacology and toxicology. According to the results of these studies, pharmaceutical formulae have been developed from plants in their natural form or in the form of infusion in ointments and syrups. These phytomedicines are called Improved Traditional Medicines. DMT has so far developed 12 ITMs that have been standardised according to traditional administration regimes. The doses have been investigated for lack of toxicity and the expiration dates for the products have been determined. These products are now being sold in drugstores in Mali. Seven of these are acknowledged as essential medicines by the Health department in Mali. These ITMs are: *Balembo *against cough, *Dysenteria*l against dysentery, *Gastrosedal *against ulcers and gastritis, *Hepatisane *against hepatitis, *Laxia-cassia *against constipation, *Malarial *against malaria and *Psorospermine *against dermatitis [2].

In this willpower to develop new traditional medicines, several ethnobotanical studies were carried out on medicinal plants from Mali [4-7]. Our study is placed within this framework.

The aim of this study was to identify different uses of certain medicinal plants which were retained for the investigations as new ITMs, but a lack of substantiated information on their use in traditional medicine made this survey necessary. Seven plants were chosen for this study and their uses in traditional medicine were investigated. In February 2005 an ethnopharmacological survey was carried out in Siby, Doila and Kolokani in nearby areas of Bamako, the capital of Mali. The village and healers in the areas to be interviewed were selected randomly and no appointment was made prior to the visits.

The result will give an overview on the cure potency of these plants according to the traditional medicinal healers in these areas. The plants will later be investigated for chemical pharmacological and toxicological aspects by DMT in order to be developed into new Improved Traditional Medicines that can be registered by the Health department in Mali.

## Methods

### Description of the study area

**Figure 1:**
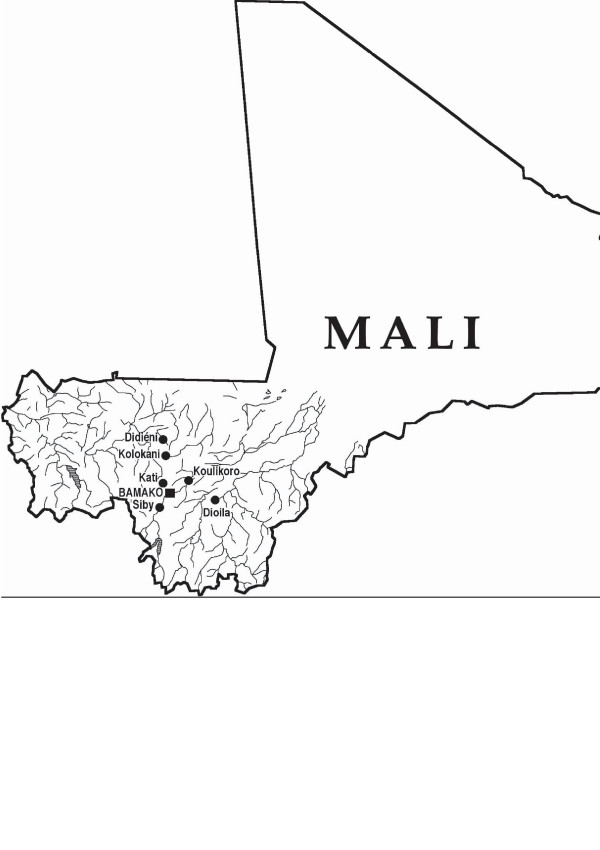
Map of Mali with focus on the survey areas.

The rural district of Siby is situated 50 km south of Bamako, Kolokani 140 km north of Bamako and Doila approximately 130 km east of Bamako. Five of the visited villages belong to the Siby region, these are: Dioulafondo, Guena, Kalassa, Djissoumala and Kakan; Five belong to the Dioila region: Dioila, Falakono, Diana, Finianan and Wolome. Didieni, Kolokani and Niamabougou belong to the Kolokani region. The location of the main areas can be seen from the map (figure 1). These areas are part of Koulikoro, one of the administrative divisions of Mali. The main income sources in these areas are agriculture and the commerce of agricultural products. Doila is one of the main areas for production of cotton in Mali. Public health services are used, but home medication is practiced primarily with medicinal plants. Traditional medicine is the first choice for the population for health problems, and healers in these areas are reputed to have good knowledge on medicinal plants and disease treatment.

### Interviews with the traditional healers

Conversations with the healers were used to obtain information on the use of the seven plants being the object for this survey. The healers that consented were asked to give their knowledge about the diseases they used those plants against, the method of preparation of the remedy, details of administration, including the approximate amounts and number of doses per day or week, the adverse effects of the remedy and how to treat these adverse effects. Traditional gifts of cola nuts and money were bestowed upon the traditional healers. The conversations were built on trust with the common goal to improve the health situation in the country and to preserve and increase the knowledge on medicinal plants.

The following seven plants were the focus for the ethnopharmacological survey: *Opilia celtidifolia *Guill. & Perr. (Opiliaceae), *Anthocleista djalonensis *A Chev. (Loganiaceae), *Erythrina senegalensis DC *(*Leguminosae: Papilionoideae*), *Heliotropium indicum *Linn (Boraginaceae), *Trichilia emetica *Vahl, (Meliaceae) *Piliostigma thonningii *Schum (Leguminosae: Caesalpinioideae) and *Cochlospermum tinctorium *A. rich. (Cochlospermaceae). The results of the survey are given in Table 2 (see additional file 1).

### Fidelity level

The fidelity level (Fl) [8] among the healers from the same district was calculated according to a following formula:

Fl (%) = (Np/N) × 100

Np is the number of healers from one given district that claim a use of a plant species to treat a particular disease, and N is the number of healers from the same district that use the plants as a medicine to treat any given disease. The formula was applied in order to compare data from different district where the survey was performed.

Below we will discuss the information given on the uses of these plants as well as to give a survey on the scientific knowledge available in the literature. The literature research was carried out for all the plants within the databases available via the library of the University of Oslo, Norway. These are: Scifinder, BIBYS, Biological abstract/WebSPIRS and OVID web.

## Results and discussion

In our study, totally 94 healers from 13 villages from the three areas were interviewed (Table 1). Most of them were members of the traditional healers association in their region and also registered by the Department of Traditional Medicine. 34% of the interviewed healers were from Siby, 35% were from Doila and 31% were from Kolokani. Men dominate the practice of traditional medicine, 76 of the interviewed healers (81%) were men over 40 years; the oldest being 104 years old. Few healers were below 40 years, amongst these, the youngest was 27. 18 women were interviewed in this study; they seem to have less knowledge than men about traditional medicine. This is because they mainly treat children and typical child diseases, while men treat both children and adults. The average age of the women is not known, most of the women have an approximate idea about when they were born, but not the exact year.

**Table 1:** An overview of the traditional healers interviewed.

Districts	Villages	Numbers of healers	Sex		Age range
			Males	Females	
Siby	Djissoumala	7	6	1	28–76
	Dioulafondo	12	8	4	30–79
	Guena	7	7	0	42–85
	Kalassa	2	2	0	35–55
	Kakan	4	3	1	65–85

Doila	Doila	6	5	1	47–70
	Falakono	12	8	4	38–80
	Diana	5	4	1	27–70
	Finianan	8	7	1	49–104
	Wòlòmè	2	1	1	56–80

Kolokani	Kolokani	15	12	3	45–73
	Didieni	9	9	0	55–62
	Niamabougou	5	4	1	50–61

The results of the survey are presented in Table 2 (see additional file 1) and discussed below.

### Medical applications of the plants

The present ethnopharmacological survey has gathered information on about 50 different diseases treated by these 7 plants (Table 2, see additional file 1). The most frequent reported ailments were malaria, abdominal pain and dermatitis. *Opilia celtidifolia *and *Trichilia emetica *were the most used plants based on the high number of uses reported. The healers consensus based on the fidelity level (Fl) index [8] was calculated for the most frequently reported diseases or ailments (Table 3). The fidelity level of the healers that use *O. celtidifolia *against malaria and abdominal pain is high in Doila (61 and 54% respectively). In Siby *O. celtidifolia *is mainly used against dermatitis (Fl = 75%) while the main use of the plant in Kolokani is against abdominal pain (Fl = 23%). *T. emetica *is mainly used against malaria in Doila and Siby (Fl = 47 and 41% respectively), while the use against abdominal pain is the most reported use in Kolokani (Fl = 32%). FI were not determined for the other plants and other uses due to the low number of reports.

**Table 3:** Comparison of the use of *Opilia celtidifolia *(*Oc*) and *Trichilia emetica *(*Te*) in the Doila, Siby and Kolokani regions based on the fidelity level.

	Fidelity level (%)					
	Siby		Doila		Kolokani	
Main reported diseases	*Oc*	*Te*	*Oc*	*Te*	*Oc*	*Te*

Malaria	17	41	61	47	22	13
Abdominal pain	23	36	54	32	23	32
Dermatitis	75	40	21	40	4	20

Below the most frequent uses of each plan is coupled with information from scientific literature.

### *Opilia celtidifolia *Guill. & Perr. (Opiliaceae)

Local Name: Korôgué

#### Traditional use

*Opilia celtidifolia *is well known to the traditional healers in our study region as a remedy to cure several diseases. The main reported disease is dermatitis (dermatitis is by the healers used as a common terminology for all kinds of skin disorders); the frequency of citations is 19.2% of 125 total citations. This use is common in Siby were the plant has a reputation for curing skin lesions and wounds; followed by malaria (14.4%), another frequent disease reported by the healers in the southern part of Mali where the survey was carried out. *O. celtidifolia *is known in the district of Doila as an appetizer (10.4% of frequency of citation), as an abdominal pain killer (10.4%) and an intestinal worm cure (7.2%); this last use was reported several times in Kolokani as well. Various other ailments were also reported (Table 2, see additional file 1).

According to the literature, decoction of the leaves is used as febrifuge in Ivory Coast. In Senegal it is used as a gargle, against dental abscesses, to treat oedema leprosy, acting as a purgative, and used against headache. A macerate left to stand overnight and strongly salted, taken on an empty stomach is meant to be particularly effective in expelling oxyuris worms from children [9].

#### Biological activities

Little is found on the biological activities of *O. celtidifolia *in the literature. Shihata et al. [10] isolated saponins from the methanol extract and found antispasmodic and anthelmintic activities for these compounds. The authors also signalled the lack of information regarding the biological properties and possible therapeutic value of the plan. The effects shown above may explain both the use as an abdominal pain killer and against intestinal worms as found to be common uses in our survey.

### *Anthocleista djalonensis *A Chev. (Loganiaceae)

Local Name: Fartanlafla

#### Traditional use

The uses of this plant were reported by traditional healers in Siby only. Malaria and abdominal pain are the most frequent reported ailments with 32% of citation frequency for each. The other uses of this plant were only reported once (Table 2, see additional file 1).

According to the literature, the decoction of the leaves is in Sierra Leone drunk as a treatment against jaundice. In Ivory Coast the root is used as a diuretic and a vigorous purgative, and also as a poison-antidote, against leprosy, as an emmenagogue and in the treatment of oedemas and elephantiasis of the scrotum. The root decoction is taken against chest pains, for constipation and against gonococci [11].

#### Biological activities

In an evaluation of an extract of *A. djalonensis *for activity against bacterial isolates from cases of non gonococcal urethritis performed by Okoli et al. [12], the cold water and ethanol extract of the roots showed a remarkable broad spectrum activity against *Staphylococcus aureus *and *Escherichia coli*. Thus, the antibacterial activity exhibited by the extracts against theses organisms justifies their general use of the plant in the treatment of sexually transmitted diseases and the folklore use of aqueous decoctions of the plant in the treatment of dysentery and other gastrointestinal diseases.

### *Erythrina senegalensis *DC (Leguminosae: Papilionoideae)

Local Name: N'tékissè

#### Traditional use

This plant is not well known to the healers in Siby. Only a few indications have been reported among which the use against amenorrhoea, urinary bilharzias and sterility are the most frequent mentioned (Table 2, see additional file 1).

According to the literature, in Gambia and Senegal the sap from the crushed leaves is applied to wounds for two or three days to promote healing. In Ghana and Nigeria the pounded bark and leaves are taken by women in a soup against barrenness. They are also used as enemas. In Mali the decoction of leaves is used to provoke diuretic activity and is taken against urinary bilharzia. In Senegal a macerate of the trunk-bark is taken internally for amenorrhoea and externally against headaches and eye-troubles. In Ivory Coast the wood is chewed as an aphrodisiac [9].

#### Biological activities

The suppressive activity of *E. senegalensis *water extract of the bark against *Plasmodium berghei *in mice has been evaluated by Saidu et al. [13]. Doses of 50 and 100 m*g*/kg reduced the mean parasitemia, but the effects were not significant. The suppressive effect were 16.5 and 23.2 % respectively, while the reference standard (chloroquine) produced a profound suppression effect of 95.8%

The analgesic effect of *E. senegalensis *was examined by the same author [13]. The water extract of the bark (50–100 mg/kg) significantly inhibited acetic acid induced abdominal constriction in mice in a dose dependant manner.

In a screening for antibacterial activity by Kone et al. [14], *E. senegalensis *root ethanol extract was found to contain active bactericides with IC_50 _values of 12 μg/ml against *Staphylococcus aureus*, *Enterococcus faecalis*, *Bacillus subtilis *and *Streptococcus pyogenes*. These studies have no relevance to the uses of the plant in Mali and such studies are needed.

### *Heliotropium indicum *Linn (Boraginaceae)

Local Name: Nonsikou

#### Traditional use

In Siby the use against vomiting is the most frequently reported. Other reported uses are against amenorrhoea, baby thinness, ocular infections and high blood pressure (Table 2, see additional file 1).

According to the literature, the use of a decoction of the leaves is recorded in Sierra Leone for washing new-born babies. In Nigeria and Ghana the sap is applied to gumboils, to clean ulcers and to cure eye infections. In Guinea the decoction of the whole plant is taken as a febrifuge. In Senegal the leaf powder is applied to dermatitis and especially to suppurating eczema and impetigo in children [15]. The leaf decoction is used in Indonesia for thrush and in poultices for herpes and rheumatism. In Ivory Coast the dried leaf powder is taken up by the nose as decongestant in colds and sinusitis [16]

#### Biological activities

Wound healing activity has been reported by Reddy et al. [17]. They showed that topical application of 10% w/v of *H. indicum *increased the percentage of wound contraction and completed wound healing by 14^th ^day indicating rapid epithelization and collagenization. The control used healed a similar wound in 23 days. An increase of the tensile strength indicated the increase in collagen facilitating wound healing.

Kugelman et al. [18] isolated the N-oxide of the alkaloid indicine from *H. indicum *and observed significant anti-tumour activity of the compound in W-256 carcinosarcoma, L-1210 leukemia, P-388 leukemia, P-1534 leukemia and melanoma B-16 tumour systems. On the basis of these results the compound was selected for human clinical trials. Studies related to the uses in Mali have not been performed.

### *Trichilia emetica *Vahl, (Meliaceae)

Synonym: *Trichilia roka *Chiov.

Local Name: Soulafinzan

#### Traditional use

Several uses were reported for *T. emetica*, the main according to the citation frequency is against malaria 23.8%. This use is a common knowledge wherever the survey was carried out. The next most frequent use is against abdominal pain (19.2%), against dermatitis (7.7%), haemorrhoids (6.2%), and jaundice and chest pain (5.4%). Several other diseases were also reported for the use of *T. emetica *(Table 2, see additional file 1).

According to the literature, in eastern Africa the root bark decoction is used as an emetic and a purgative, against fever, epilepsy, leprosy and makes women fecund. In Senegal the leaf decoction is used against blennorrhoea; the infusion against headache and as lotion on burns [9]. The leaf decoction is used against malaria and scabies; the stem and leaf decoction is used against intestinal, coetaneous or mouth infections. The fruit is in eastern Africa used as a diuretic. *T. emetica *is also used against poisoning, hepatitis, ulcer, dysmenorrhoea, asthma, cirrhosis and internal worms [19].

#### Biological activities

In the exploration of biological activities, the family Meliaceae has attracted extensive attention. *T. emetica *particularly has been largely investigated. El Tahir et al. [20] investigated the anti-plasmodia activity of the methanol extract of the leaves and found an IC_50 _of 2.5 μg/ml against *Plasmodium falciparum *sensitive strain Dd2 and 17.5 μg/ml against the resistant strain 3D7. Anti-inflammatory activity was demonstrated by McGaw et al. [21]; they found an inhibition of 89% of prostaglandin synthesis by the ethanol extract of the plant. Sanogo et al. [22] demonstrated the antipyretic activity, which confirmed the traditional use of this plant as an antipyretic agent. The complement activating effect was investigated by Diallo et al. [23] that found the leaf water extract to have an effect on the complement system with an IC_50 _of 45 μg/ml which may be related to the healing of burns and wounds.

### *Piliostigma thonningii *Schum (Leguminosae: Caesalpinioideae)

Local Name: Niama

#### Traditional use

According to the traditional healers in Doila this plant is call "child remedy'' as it is mainly used as a remedy for children. The different indications of this plant are almost all related to children, except its use against arthritis, headache, haemorrhoids and backache. The most frequent of them according to the citation frequency is the use against malaria (40%) followed by the use against the children digestive disorder called abdominal flatulence (16%) and child malnutrition (8%). According to the local traditional beliefs,"preparation from *P. thonningii *is the first plant remedy given to a child in his life".

In the literature, the most frequent use of the bark of *P. thonningii *is in treating cough, usually as an infusion or by chewing of the bark. A common use in Uganda is to stop diarrhoea, dysentery and intestinal upsets. The bark infusion or maceration also enters into the treatment of malaria and leprosy; analgesic properties are described to the bark; preparations are also used for sore throat, toothache, stomach-ache and earache [24].

The leaf decoction is a laxative and is given to children [25]. The infusion is given to new born babies and used as a tonic embrocating to massage the mother's abdomen; it serves also as a lotion for lumbago. The leaves, after soaking in hot water, are applied topically as wound-dressing and a leaf decoction is applied to the excision wounds in the South-West Africa region [11].

#### Biological activities

Asuzu et al. [26] found that the D-3-O-Methylchiroinositol, the anthelmintic component of *P. thonningii *stem bark extract, induced approximately 60% larval paralysis within 24 h of contact with *Haemonchus contortus *larvae at 4.4 mg/ml. This level of activity confirms the use of *P. thonningii *stem bark extract to treat helminthiasis in African traditional medicine.

Akinpelu et al. [27] founded that *P. thonningii *stem bark extract, at a concentration of 20 mg/ml, exhibited an antibacterial activity against *Bacillus subtilis*, *Staphylococcus aureus*, *Shigella dysenteries*, *Escherichia coli *and *Proteus ulgaris*.

### *Cochlospermum tinctorium *A. Rich. (Cochlospermaceae)

Local Name: N'Tiribara

#### Traditional use

According to our ethnopharmacological survey, this plant is mainly used against jaundice (42.4%) based on its citation frequency. It is also used against malaria (27.3%). The uses against abdominal pain, in wound healing, haemorrhoids, intestinal worms, bilharzias and hepatitis were also reported (Table 2, see additional file 1).

These results are quite similar to those found by Nergård et al. [7] during another survey performed on *C. tinctorium *in another area in Mali. In addition to the ailments mentioned above it was also reported to be used against gastrointestinal diseases such as ulcer and stomach ache, flatulence and constipation.

In Ivory Coast the root is used for oedematous conditions, for orchites, schistosomiasis, jaundice, fevers, epilepsy, pneumonia, intercostal pains, and bronchial affections, in eye instillations for conjunctivitis and for indigestion and stomach pain [28].

#### Biological activities

The ethanol extract of the root of *C. tinctorium *showed a pronounced activity against a chloroquine-sensitive (3D7) and -resistant (Dd2) *Plasmodium falciparum *strain with an IC_50 _of 2.3 and 3.8 μg/ml respectively [29].

The aqueous extract, ethanol and hydro-ethanol extract of the root of *C. tinctorium *significantly inhibited the toxic effect of tert-butyl hydro peroxide-induced malonaldehyde formation in isolated rat hepatocytes at doses equivalent to 1 mg of dried plant material per ml of cell suspension. They exhibited marked effect against induction of lipid peroxidation and hepatocyte lysis. This result showed the hepatoprotective effects of these extracts [30].

In the anti ulcer test Nergård et al. [7] found that the oral administration of 25, 50 and 100 mg/kg of the crude extract of *C. tinctorium *1 h before the HCl/Ethanol treatment significantly reduced the occurrence of mucosal gastric lesions in mice. This result confirms the reported use against gastro-intestinal diseases by the traditional healers.

### Plant parts used and mode of preparation

The leaves are the most frequently used plant part (56.3%), the root and fruits are used about 30% and 8.5% respectively, and the less used plant part is the bark (5.3%). In another study of plants used for wound healing in Dogonland (Mali), Inngjerdingen et al. [5] found that the leaves and the roots were the most frequently plant parts used, constituting about 22 and 24% of the preparations, respectively, followed by the stem bark and fruits (12% each). In our study the exception were for *Cochlospermum tinctorium*, almost all traditional use reported for this plant was related to the root (about 94% of the number of citation of the plant). The leaves of this plant were used for the treatment of malaria and jaundice, a result that is similar to those obtained by Nergård et al. [7] on the use of *Cochlospermun tinctorium *in Mali. The root was in that report also the part of the plant most frequently used (95%), while the leaves were used by a minority of the healers for the treatment of malaria, ulcer and flatulence, the flowers were used by only one healer in the treatment of constipation. In our study no use of flower was reported for *Cochlospermun tinctorium*.

According to one of the traditional healers, the need for the use of stem bark will increase when the leaves are not available. The study area has a wet season from June to September, the main rainy season, and a dry and windy season from February to May. During this period most of the plants do not have leaves and the use of stem bark and dry plant parts is for this reason frequent. The fruits are also used, but not often due to their short time of availability.

The majority of the remedies are prepared in the form of decoction of fresh leaves. In our study area people do usually not store remedies for prolonged period of time. When needed they go out and collect the plant and prepare the remedy from fresh or sun dried material.

The powders are prepared by pounding the fresh plant part or the crushed plant material after sun drying, in a wooden mortar.

Water is the most frequent liquid used in preparations; powders are sometimes suspended in milk or consumed in food such as porridge and sauce.

Decoction is the most frequent way of preparation of the remedies (65%) followed by infusion (13%), which is used for the powders; the maceration (11%) is mostly used for the root preparation. Some remedies are prepared from a single plant species, but in a few cases mixtures of plants or other substances are added as can be seen from Table 2 (see additional file 1).

### Route of administration and dosage

Most of the remedies are taken orally and by external application as body bath, steam bath, and as ointment in the case of dermatitis. Some remedies used for the treatment of haemorrhoids and genital infection are used as enema. Fumigation is mainly used in the treatment of headache and chest pain.

According to some healers certain additives are frequently used to improve the acceptability of some remedies that are taken orally. This can be salt, which is often mixed with powder; sugar or honey is added to decoctions and macerations to reduce the bitterness of the remedies in order to make them easer to drink. Lack of data on the biological role of these additives has been notified in the literature. The plants cited in the remedies are generally evaluated by biological screening, but the additives mentioned by the healers are normally not investigated.

Some healers reported that restrictions are imposed when certain types of remedies are taken by patients. For example, food is not given from the morning until noon to a patient who is taking a remedy against intestinal worms. This is the estimated time for getting diarrhoea which expulses the worm from the intestine. It is believed that food will reduce the efficacy of the remedy.

For most of the remedies, the dose given to the patient depends on age, physical and health condition of the patient, and the duration of the illness. The doses vary from a teacup (70 ml) for adults to a handful (25 ml) for a child; a lack of agreement among the healers on doses of remedies was sometimes noted. The variation of the doses from one healer to another may show that the plants have a low degree of toxicity. For pharmacological investigation the active doses of these plants may not be high since they appear to treat the patients with low doses. The duration of treatment is not given for all remedies. According to the healers duration of treatment is difficult to determine and depends on how long the patient has been ill. The patient is supposed to take the remedy until healed, and then, the only one able to determine the end of a treatment is the patient himself since the remedy is taken at home in the absence of the healers.

### Adverse effects

The reported adverse effects for the use of these seven medicinal plants are diarrhoea, vomiting and dizziness. According to the healers these effects are generally due to an overdose of the remedy. Sometimes the expected effect of the remedy is diarrhoea, such as in the cases of constipation and intestinal worms. For intoxication treatment, the patient is supposed to eliminate the poisons by vomiting. In a survey of toxic plants on the market in the district of Bamako [31], *Trichilia emetica *was reported to be a toxic plant. The toxic effects reported were diarrhoea and vomiting. In our study no toxic effect was reported, but the same symptoms (diarrhoea and vomiting) were qualified as adverse effects for some remedies. According to the healers the adverse effects are generally moderate, and disappear at the end of the treatment. The commonly used remedy against diarrhoea and vomiting is a cold bath; another plant part can be used when the adverse effect is violent and stopping of the treatment is recommended.

## Conclusion

Our ethnopharmacology survey showed that medicinal plants are still widely used by the population in the area where the study was conducted. It allowed us to report 50 different diseases or ailments treated by the seven medicinal plants included in this survey. Several types of preparations of these plants were used. The plants grow over an extended area and are used by healers separated by long distances. This may explain the many different types of uses observed. The healers' consensus in the treatment of the main reported diseases is fairly high, giving an additional validity to the plants as a traditional remedy.

This study complements the on-going activities of evaluation of different uses of medicinal plants and the development of new Improved Traditional Medicine by the Department of Traditional Medicine in Mali. Pharmacological, toxicological and phytochemical studies will be carried on these plants in order to ascertain the effectiveness as well as the possible toxicity of the remedies followed by designing therapeutic strategies based on the most effective and least toxic plants.

## Competing interests

The author(s) declare that they have no competing interests.

## Authors' contributions

AT, DD and SD performed the interviews with the healers and identified all plant material described.

AT, HB and BSP drafted and finalised the manuscript.

## Supplementary Material

Additional file 1 Click here for file
