# Supplementary material for: Ethnopharmacological survey of different uses of seven medicinal plants from Mali, (West Africa) in the regions Doila, Kolokani and Siby
Source: J Ethnobiol Ethnomed. 2005 Sep 27;1:7. doi: 10.1186/1746-4269-1-7 (PMC1277087; doi:10.1186/1746-4269-1-7)
Supplement: Additional file 1 [file 1746-4269-1-7-S1.pdf]

Table 2. Medical uses of seven different plants described by the healers in the regions of Doila, Kolokani and Siby; Mali.

Plants	Indications	Preparation and use of the remedy	Number of citations	Total Number of citations	% related to each plant
Opilia celtidifolia	Dermatitis	- a decoction of leaves is used daily as bath for 2 days.<br>- leaf decoction is used as bath and the powder as ointment on dermatitis lesions, use until healed.<br>-An infusion of leaf powder is used as bath and drunk.<br>- decoction of the leaves of *O. celtidifolia* and the stem bark of *Khaya senegalensis* is used as bath.<br>- a macerate of the roots of *O. celtidifolia* is made with some water used for washing *Shorgum vulgarae* seeds; drink one teacup of the macerate 3 times daily for 1 week.	20<br>1<br>1<br>1<br>1	24	19.1
	Malaria	-drink the leaf decoction 1-2 times daily and use as a bath for 3 days; this remedy can induce diarrhoea; which antidote is a cold bath.<br>-make a macerate of the roots of *O. celtidifolia* made with water used for washing the seeds of *Sorghum vulgaris*, drink the macerate daily.	14<br>1	18	14.4

	Neuralgic<br>Malaria	- a decoct of the leaves of *O. celtidifolia* plus the leaves of *Trichilia emetica* is made and 1 teacup drunk 2 times every day until cured, the remedy can induce dizziness, nausea and vomiting, if so stop the treatment.<br>-boil the leaves in water plus the juice of *Tamarindus indica* fruit; drink the filtrate until healed.<br>-crush the leaves in water, use the filtrate as bath.	1<br>1<br>1		
	Abdominal pain	-drink the leaf decoction and use it as bath.<br>-drink the root decoction in the morning and evening for 2 days. If diarrhoea occurs, the antidote is the powder of the fruit of *Piliostigma thonningii*.<br>-add the leaf powder to porridge and eat; vomiting can arise for this treatment.<br>-the 24h macerate of the root is drunk once daily for 2 days. This remedy can not be given to pregnant women.<br>-mix the leaf powder with *Penessetum sp* seed	7<br>2<br>2<br>1	13	10.3

		powder, use the mixture for making porridge and eat. The remedy can induce vomiting.	1		
	Appetizer	-the leaf decoction is drunk as appetizer<br>-mix the leaf powder in porridge and eat; or make an infusion with the powder and drink.<br>-make a decoction of the leaves added to the fruit of *T. indica* or add the powder of leaves to an infusion of *T. indica* fruit and drink; this treatment can induce diarrhoea	10<br>2<br>1	13	10.3
	Taeniasis and intestinal worms	- drink the leaf decoction, moderate diarrhoea can be induced by the treatment.<br>-mix the leaf powder with *Penessetum sp* seed powder, eat the mixture on an empty stomach. The treatment can induce vomiting and diarrhoea, drink porridge against the vomiting and diarrhoea.<br>-drink the leaf powder infusion.<br>-drink the root decoction or the powder infusion in the morning with an empty stomach and do not eat until noon. The treatment can induce moderate diarrhoea.	4<br>3<br>1<br>1	9	7.1
	Headaches	-use the leaf decoction as head steam bath and for washing.	2	6	4.8

		-the leaf decoction is drunk, used as steam bath and head wash once everyday for 6 days.<br>-mix the leaves with the root of *Ximenia americana*, drink the decoction and use as bath.<br>-use the leaf powder for fumigation of the head.	2<br>1<br>1		
	Tiredness	- the decoction of the leaves of *O. celtidifolia and Annona senegalensis* is used as a bath.<br>- the leaf decoction is used as bath and drunk.<br>- the leaves crushed in water is used as bath and drunk.	2<br>1<br>1	4	3.2
	Sexual Asthenia	-drink the leaves decoction until healed.<br>-eat the leaf powder.<br>-drink the decoction of the leaves of *O. celtidifolia* and the fruit of *Xylopia aethiopica* with sugar, in the evening.<br>-t he leaves of *O. celtidifolia*, the *Lorantus sp* of *Khaya senegalensis* and the fruit of *Borassus aetiopum* are burnt and pound, the powder is mixed in the sauce and eaten until cured.	1<br>1<br>1<br>1	4	3.2
	Chest pain	-the leaf decoction is used as a steam bath and as bath.	2	3	2.4

		-the leaf powder is used for fumigation of the chest 2 times daily for 6 days.	1		
	Wound healing	-wash the wound with the stem bark decoction of *Terminalia sp* and apply the fresh leaves crushed or the leaf powder of *O. celtidifolia.*<br>-apply the powder of the leaves of *O. celtidifolia* and *Guiera senegalensis* to the washed wound.<br>-apply the concentrated decoction of the leaves to the washed wound until healing.	1<br>1<br>1	3	2.4
	Intoxications	- a leaf decoction, is drunk and the filtrate used as bath one time daily for 3 to 4 days; in case of diarrhoea have a cold bath.<br>- a decoction with the leaves of *O. celtidifolia* and the roots of *Swartzia madagascariensis* is made and drunk until healed	1<br>1	2	1.6
	Nightmare	-the bark powder is used as ointment and fumigation at bed time.<br>- drink the leaf decoction.	1<br>1	2	1.6
	Constipation	-the root powder is mixed with curdled milk and drunk. 1 or 2 times are enough to cure constipation.<br>-drink the leaf decoction.	1<br>1	2	1.6
	Urinary calculus	-the concentrated decoction of *O. celtidifolia* and *Tamarindus indica* leaves or fruit is drunk 2	2	2	1.6

		times daily for 7 days.			
	Dizziness	-drink the leaf decoction and use it as a bath.	2	2	1.6
	Child Paralysis	-use the leaf decoction for washing and rubbing the feet until healed	2	2	1.6
	Cataracts,<br>Eye infection	-use the decoction of the leaves of *O. celtidifolia* and *Tamarindus indica* for washing the eyes 2-3 times daily for 15 days.<br>-use the leaf decoction as eye wash.	1<br><br>1	2	1.6
	Gastric Ulcer	-drink the leaf decoction and use it as bath.	2	2	1.6
	Jaundice	-drink a decoction of the leaves and use it as bath until cured.<br>-eat the powder of the leaves of *O. celtidifolia* and *Striga sp* in the morning and evening until healed.	1<br>1	2	1.6
	Leprosy	-the leaf decoction is used in a bath with butter soap.	1		
	Diarrhoea	-drink the leaf decoction and use it as bath.	1		
	Genital infection	-use the leaf decoction as enema until healed.	1		
	Asthma	-the leaves of O*pilia* are mixed to the *loranthus* of *Manelikara multiverdis*, the decoction of both is drunk, used as bath and steam bath 2 times daily for 4 days.	1		
	Bite of snake or dog	-use the leaf decoction for washing and drink.	1		
	Indigestion	-drink a root decoction in the evening.	1		

	Thinness	-the leaf decoction is used as bath and drunk.	1		
	Cold	-the leaf decoction is used as steam bath of the head and the chest and is drunk.	1		
	Amenorrhoea	-the decoction of the leaves of *O. celtidifolia* the roots of *Cochlospermum tinctorium* and *Exalobus monopetalus* is drunk, this treatment can induce moderate diarrhoea.	1		
	Female Sterility	-drink the root decoction, 1 glass every day until cured.	1		
	Spontaneous abortion	-add the leaf powder to porridge and eat.	1		
Anthocleista djalonensis	Malaria	-the maceration of root is drunk alone or with honey.<br>-the leaf decoction is drunk<br>- the root decoction added to honey is drunk.<br>-drink an infusion of the root powder.	4<br><br>1<br>1<br>1	7	31.8
	Abdominal Pain	-the root of *A. djalonensis*, the stem bark and the leaves of *Lansonia inermis* are mixed, the 2 days macerate of these ingredients is drunk for 7 days.<br>-drink he root decoction, this remedy can induce diarrhoea which can be cured by porridge consumption.<br>-mix the roots powder into the porridge and eat; or drink a salty root powder infusion.<br>-drink one teacup of the 24 h macerate of the	2<br><br><br><br>2<br><br><br>2	7	31.8

		roots of *A. djalonensis* and *Swartzia madagascariensis* plus honey every day until healed.	1		
	Intoxication	-drink some milk to which leaf powder is added.	1		
	Amenorrhoea	-the powder of stem and root bark plus salt is eaten, or the decoction of both is drunk.	1		
	Sexual Asthenia	-the powder of stem or root bark plus salt is added to porridge or sauce and eaten.	1		
	Teniasis	-make a decoction of roots, add honey and leave it for 7 days of maceration. -drink one glass two times daily, this remedy can induce diarrhoea.	1		
	Fever	the decoction of leaves is drunk and used as bath.	1		
	Jaundice	-drink one teacup of the macerate of the roots of *A. djalonensis* and *Swartzia madagascariensis* with honey every day until healed.	1		
	Haemorrhage	-the leaf decoction is used as steam bath and a bath.	1		
	Pregnancy Vomiting	-drink the leaf decoction 3 times daily until healed.	1		
Erythrina senegalensis	Amenorrhoea	-drink the leaf and flower decoction and use as bath.<br>-drink and use the root decoction as enema to cure menstrual disorders in women	2<br>1	3	27.3
	Sexual Asthenia	-eat the salty root powder until healed.<br>-chew the fresh branch and use as tooth brush to	1<br>1	2	182

		cure sexual impotency.			
	Urinary Bilharzias	-drink a decoction of stem bark.	2	2	18.2
	Children dentition	-the leaf decoction is used as bath and given orally to children to cure diseases related to teething.	1		
	Malaria	-the leaf or root decoction is drunk and used as bath for 3 days.	1		
	Infertility	-drink root decoction one time daily for 20 days.	1		
	Dizziness	-leaf decoction is drunk and used as bath	1		
	Vomiting	-the leaf decoction can be drunk instead of the root decoction.<br>- the root decoction is drunk for 1-2 days<br>-drink the flower decoction.	2<br>1<br>1	4	40
Heliotropium indicum	Amenorrhoea	-the root decoction is used as bath and drunk 3 days.<br>-the decoction of the leaves and the stem bark of *Parkia biglobosa* is drunk at night for 7 days.	1<br>1	2	20
	Malaria	-the leaf decoction is drunk.	1		
	High Blood-Pressure	-drink the leaf decoction.	1		
	Baby Thinness	-drink the leaf decoction and use as bath 4 times daily for 10 days.	1		

	Ocular infections	-use the leaf decoction for washing the eyes.	1		
Trichilia emetica	Malaria	-use the leaf decoction as bath and drink for 3-7 days; the decoction can induce dizziness when administrated in the morning on an empty stomach, also vomiting and diarrhoea. Drink lemon juice against the dizziness.<br>-drink the stem bark decoction with honey and use as bath until healed.<br>-drink the root decoction and used as a bath, sugar can be added to the decoction for drinking to make it more tasteful.<br>-drink the root powder infusion.<br>-the root is macerated for 7 days, the macerate is drunk for 3 days, or the root is macerated for 3 days with honey and drunk until healed.<br>-drink an infusion of powdered stem bark and leaves.<br>-drink the leaf and root decoction 2-3 times daily for 3 days.<br>-the 10 days macerate of the root from *T. emetica, Pseudocedrala kotschii, Cassia*	18<br>3<br>2<br>2<br>2<br>1<br>1	32	24.2

		sieberiana, Nauclea latifolia mixed with honey is drunk for 10 days.<br>- a 3-4 hours decoction of ~ 5 Kg of dried stem bark plus the juice of 100 lemons and 10 litres of water is drunk, 1 teacup everyday until healed.<br>-drink the decoction of *T emetica* and *Mitragyna inermis* stem bark 3 times every day until healed.	1<br>1<br>1		
	Abdominal pain	-drink an infusion or decoction of the root or the leaf powder.<br>-drink a leaf decoction and use it as bath.<br>-drink an infusion of root powder mixed with salt in morning and evening.<br>-drink the stem bark decoction or the powder infusion and use as bath until healed. Moderate diarrhoea can occur during the treatment.<br>-the root bark is crushed and dried with lemon, the powder infusion is drunk.<br>- drink the decoction of the root of *T. emetica* and those of *Tamarindus indica* the morning and evening until healed; moderate diarrhoea can arise during the treatment.	9<br>6<br>3<br>3<br>1<br>1	25	18.9

		-drink a macerate of *T. emetica* root and *Prosopis africana* stem bark, 1 teacup daily for 5 days, in case of diarrhoea have a cold bath.<br>- drink the root macerate with honey for 5 days. The overdose of the remedy can induce diarrhoea.	1<br>1		
	Dermatitis	-use the leaf decoction as steam bath, as bath and drink.<br>-apply the leaf powder on dermatitis lesions and use it for fumigation, or use the powder infusion for washing the lesions.<br>-apply the powder from the bark or the root burnt to a cinder on the lesion washed with the bark decoction.	6<br>2<br>2	10	7.6
	Haemorrhoid	-the root bark is crushed and dried with black pepper ginger, and the fruit of *Xylopia aethiopica*, infusion of the powder is drunk every day until healed. Diarrhoea and vomiting can arise during the treatment, if so drink a decoction of *Strychnos spinosa* stem bark against this.<br>-the root powder macerate in salt water is used as enema, or drink the root decoction.<br>-use a macerate of root powder of *T. emetica* and *Securidaca longepedonculata* as enema for 3	3<br>2	8	6.1

		days. The remedy will induce diarrhoea which is its good effect.<br>-crush and dry the root bark of *T emetica* and *Pseudocedrela kotschii*, use the infusion of the powder as enema.<br>-drink the infusion of root powder or the decoction of the root 3 times daily before eating.	1<br>1<br>1		
	Jaundice	-drink a leaf decoction for 7 days.<br>-drink a concentrated decoction of root; or a root powder infusion until healed.<br>-crush the root, add 2 litres of honey and 4 litres of water used for washing *Sorghum vulgaris* seeds, macerate for 24 h and drink 3 times daily until healed.<br>-drink the root decoction for 15 days.	3<br>2<br>1<br>1	7	5.3
	Chest pain	-drink the leaf decoction and use it as a bath, a steam bath and make chest massage with the decocted leaves.	7	7	5.3
	Gastric ulcer	-crush the root bark with salt and ginger, consume the powder in porridge 2 times every day until healed, or take an infusion of the powder.	2	4	3

		-drink an infusion of root powder until healed or consume the powder into porridge for 1 month.	2		
	Digestive infection	-mix the salty root powder to sauce, coffee or porridge and consume.<br>-drink the 3 days root maceration for 1 month.<br>-drink a leaf decoction; it can induce moderated vomiting and diarrhoea.<br>-make a decoction of the root of *T emetica* and the root of *Cassia sieberiana,* macerate the filtrate with honey for 5 days, and drink one teacup every morning on an empty stomach until healed.	1<br>1<br>1<br>1	4	3
	Female Sterility	-drink the leaf decoction of *T. emetica* and *Combretum molle* to induce fertility and conceiving.<br>- consume the root powder added to ginger and salt in porridge to cure female sterility and its related stomach pain.<br>-drink the root and seed decoction to induce fertility.<br>- drink the leaf powder infusion made by milk.	1<br>1<br>1<br>1	4	3
	Headache	-drink the leaf decoction and use as head wash.<br>-use the leaf powder for fumigation.	3<br>1	4	3
	Thinness	use the decoction of leaves as bath and drink	3	3	2.3

		alone or with sugar added.			
	Intoxication	-drink a macerate of root powder in milk, this remedy can induce diarrhoea and vomiting, if so take a cold bath to stop these symptoms or consume some porridge.	3	3	2.3
	Tiredness	-use a leaf decoction as bath.<br>-Use the root powder as ointment and as a body massage.	2<br>1	3	2.3
	Leprosy	-drink the root decoction and use it as bath.<br>-drink the leaf decoction and use as bath until healed.	1<br>1	2	1.5
	Urinary infection, bilharzias	-drink the decoction of the leaves and the fruits of *Sticnos spinosa* until healed.<br>-drink a root powder infusion.	1<br>1	2	1.5
	Dysmenorrhoea	-use the leaf decoction of *Lorantus sp* from *Vitex doniani* and *T. emetica* as enema to stop bleeding after delivery.<br>-drink a decoction of the leaves of *T. emetica* and *Tamarindus indica* or *Citrus limonene* for 7 days.	1<br>1	2	1.5
	Constipation	-drink a leaf or a stem bark decoction for 3 days.<br>-drink the 7 days root macerate for 3 days.	1<br>1	2	1.5
	Abdominal flatulence	-drink the leaf decocts of *T. emetica* and *Opilia celtidifolia.*<br>-drink an infusion of *Acacia nilotica* seed powder	1<br>1	2	1.5

		mixed to *T. emetica* root powder.			
	Ensorcelled	-use the leaf decoction as bath and drink.<br>-Use the root maceration as bath and drink.	1<br><br>1	2	1.5
	Teniasis	-crush and dry the stem bark with the root of *Securidaca longepedonculata,* drink the macerate of the powder once daily for 1-3 days. Diarrhoea and vomiting can arise during the treatment.	1		
	Pneumonia	-drink and use as bath the leaf or root decoction for 12 days.	1		
	Swelling	-apply the root powder mixed with potassium hydroxide dissolved in little water.	1		
	Eye infection	-use the leaf and stem bark decoction as eye wash 2 times every day.	1		
	Cough	-drink a leaf decoction.	1		
	Breast pain	-use the leaf decoction as breast steam bath.	1		
Piliostigma thonningii	Malaria	- drink and use as bath the leaf decoction.<br>-drink and use as bath the leaf powder macerate.<br>-use a decoction of *P. thonningii* and *Guiera senegalensis* leaves as bath and drink.<br>-the stem bark decoction is drunk and used as bath.	7<br><br>1<br><br>1	10	40

			1		
	Abdominal flatulence	-drink the leaf decoction.	4	4	16
	Malnutrition	-drink a leaf decoction.	2	2	8
	Dysentery	-consume the powder of the fruit mixed with salt.	1		
	Arthritis	use the powder of the fruit as ointment on joints.	1		
	Headache	-use the dried fruit for fumigation of the head.	1		
	Cough	-drink an infusion of the mixture of the stem bark powder from *P. thonningii* and *Anogeusus leiocarpus*.	1		
	Wound healing	-use the root decoction for washing the wound and apply the powder of the root burnt to a cinder.	1		
	Haemorrhoid	-consume the powder of the fruit mixed with salt.	1		
	Jaundice	-use a leaf decoction as bath and drink.	1		
	Backache	-use the leaf decoction as bath.	1		
	Thinness	-consume the leaf powder in food.	1		
Cochlospermum tinctorium	Jaundice	-drink an infusion of root powder.<br>-consume the root powder in porridge.<br>-use the root macerate as bath and drink 7 times the same day or drink the root macerate with honey until healed.	4<br>3<br>3	14	42.4

		-boil eggs in the root decoction, use the decoction as bath and consume the eggs; the remedy can induce vomiting.<br>-use the root decoction for coking sauce or meat and eat.<br>--drink the root powder dissolved in the juice of *Tamarindus indica* fruit.<br>-drink an infusion of leaf powder added to honey at morning and evening for 7 days.	1<br><br>1<br><br>1<br><br>1		
	Malaria	--drink an infusion of the root powder or consume the root powder in porridge.<br>-use the leaf decoction for cooking sorghum which is used as meal.<br>-drink the decoction of the root of *C. tinctorium* and *Swartzia madacasriensis* 2 times daily for 5 days.	7<br><br>1<br><br>1	9	27.3
	Abdominal Pain	-consume eggs containing the root powder, or drink a root powder infusion 3 times every day until healed.<br>-drink an infusion of root powder in the morning on an empty stomach.	1<br><br>1	2	6.1
	Haemorrhoid	-consume sauce or porridge cooked with the root decoction 2 times weekly in 2 weeks.	1		
	Bilharzias	-consume porridge containing the root powder 2 times daily for 2 days.	1		
	Hepatitis	-consume the root powder mixed with onion and	1		

		garlic in porridge.			
	Teniasis and other intestinal worm	-make an infusion with the powder of the root crushed and dried with salt and the fruit of *Xylopia aethiopica*, drink in the morning on an empty stomach.	1		
	Snake bite	-the root macerate induces vomiting which eliminate snake poison.	1		
	Dysmenorrhoea	-drink the 24 hours root powder macerate.	1		
	Wound	-apply the root powder on the wound.	1		
	Pneumonia	-drink an infusion of root powder.	1		

The values of the percentage for the remedies reported only once are not given.
